# Strength Prediction of Cement-Stabilized Steel Slag Using Deep Learning and SHAP Analysis

**DOI:** 10.3390/ma19040795

**Published:** 2026-02-18

**Authors:** Zunqing Liu, Yifei Wang, Jian Sun, Haojie Ji, Xiaoman Shan, Fei Liu

**Affiliations:** 1 School of Traffic and Logistics Engineering, Xinjiang Agricultural University, Urumqi 830052, China; wangyifei808@163.com (Y.W.); sunjian2021@gmail.com (J.S.);; 2 Key Laboratory of Transportation and Logistics Engineering in Xinjiang, Urumqi 830052, China; 3 College of Hydraulic and Civil Engineering, Xinjiang Agricultural University, Urumqi 830052, China; 4 Xinjiang Production and Construction Corps Survey and Design Institute Group Co., Ltd., Urumqi 830002, China

**Keywords:** steel slag, deep learning, cement-stabilized steel slag, SHAP analysis, mechanical properties

## Abstract

This study combined experimental analysis with deep learning to investigate the effects of curing age, steel slag content, and gradation composition on the mechanical properties of cement-stabilized steel slag (CSSS). The strength evolution patterns and underlying microscopic mechanisms were systematically elucidated. Experimental results showed that CSSS strength grows nonlinearly with curing age, with optimal mechanical performance achieved at a 60% steel slag content. The microstructural evolution characterized by SEM-EDS and XRD revealed that steel slag incorporation promotes the formation of AFt and densifies the gel network. In later curing stages, natural carbonation of Ca(OH)_2_ and secondary hydration of reactive steel slag components produce CaCO_3_ and additional C-S-H gel, which fill pores and significantly enhance long-term strength. A CNN-GRU-Attention model was developed to predict the unconfined compressive strength (UCS) and splitting tensile strength (STS) of CSSS. In a single data split, the model achieved R^2^ values of 0.9875 for UCS and 0.9911 for STS, with RMSEs of 0.2577 MPa and 0.0234 MPa, and MAEs of 0.2059 MPa and 0.0184 MPa, outperforming all benchmark models. Under rigorous 5 × 5 repeated cross-validation, it maintained the highest average R^2^ (UCS: 0.9417, STS: 0.9329) and the lowest error metrics, confirming its robustness and generalization capability. SHAP and Pearson correlation analyses identified cement content as the primary strength determinant, while steel slag content exhibited a threshold effect, highlighting the importance of prudent gradation control in practical engineering. This study provides both a theoretical foundation and a methodological framework for analyzing variable interactions and predicting the strength development of CSSS.

## 1. Introduction

Cement-stabilized materials are extensively used as the primary load-bearing layer in China’s high-grade highway pavement structures, accounting for over 85% of applications [1,2]. These materials provide advantages such as high strength, excellent integrity, robust stability, and economic feasibility, making them the predominant material for road base course [3]. However, as a semi-rigid base material, it still suffers from problems such as low flexural tensile strength and poor crack resistance. Under external influences such as sudden temperature drops or moisture variation, they are prone to shrinkage cracking, which can propagate as reflective cracks into the asphalt concrete pavement layer, compromising the durability and serviceability of the pavement structure [4]. This problem is particularly exacerbated in the extreme climatic conditions of frigid, arid regions such as Xinjiang, severely limiting pavement service life [5,6,7]. Compounding these technical challenges, the ongoing advancement of the “dual carbon” goals and increasing restrictions on natural aggregate extraction have made the pursuit of high-performance, environmentally friendly alternative materials an urgent industry need [8].

With the rapid expansion of China’s steel industry, annual steel slag output has surpassed 100 million tons. However, its overall utilization rate remains below 30% [9,10,11], leading to substantial resource waste and significant environmental burdens from land occupation and pollution [12]. Due to its high hardness, strong abrasion resistance, and potential cementitious activity, steel slag exhibits considerable promise as a substitute for natural aggregates in construction [13,14,15,16,17,18]. The mineral components in steel slag, such as C_2_S and C_3_S, are similar to those in cement and possess certain hydration characteristics, which can significantly improve the macroscopic mechanical properties of semi-rigid base materials [19,20]. Yang et al. [21] incorporated steel slag into cement-stabilized macadam and, through analysis of its hydration characteristics, microstructure evolution, and strength development patterns, found that the addition of steel slag improved the overall strength of the mixture. Huang et al. [22] confirmed that steel slag outperformed natural crushed stone in density, abrasion resistance, and fracture resistance, and determined that mechanical properties peaked at a 50% slag content, beyond which increased porosity led to strength reduction. Similarly, Liu et al. [23] demonstrated that a 50% steel slag content yielded the optimal strength, improved resistance to freeze–thaw cycles and dry shrinkage, and reduced thermal shrinkage. Their SEM and XRD analyses revealed markedly increased hydration products, a denser cementitious matrix, and lower porosity at this optimum dosage. Feng et al. [8] further showed through mechanical tests that steel slag–modified materials exhibited over 50% higher compressive strength than conventional cement-stabilized materials. In particular, materials prepared with oxalic acid-treated steel slag exhibited superior mechanical properties and significantly extended service life. However, in addition to the cementitious active components, steel slag also contains a large amount of inert components, represented by the RO phase (solid solution of MgO, FeO, and MnO) and Fe_3_O_4_ [24,25]. These inert components often involve slow physicochemical processes and long-term interactions with the environment [26], resulting in complex development patterns. Furthermore, improving mechanical properties with these inert components typically requires long curing periods. Verifying their performance and elucidating the underlying mechanisms necessitate long-term experiments and continuous microscopic observations, leading to lengthy research periods and high costs. This presents challenges for predicting the performance and application of steel slag in practical engineering projects.

In recent years, the rapid advancement of artificial intelligence technologies has enabled the extensive application of various deep learning and machine learning algorithms—including backpropagation (BP) neural networks, random forests (RFs), long short-term memory (LSTM) networks, and convolutional neural networks (CNNs)—in predicting the properties of building materials, demonstrating significant modeling and analytical potential [27,28,29,30,31]. For example, Yu et al. [32] developed an interpretable CNN-LSTM model with an attention mechanism to predict the compressive strength of rubber-modified concrete, achieving R^2^ values of 0.967 and 0.943 on the training and test sets, respectively. Sensitivity analysis using SHAP further indicated that coarse aggregate content, rubber content, and curing time were the primary factors affecting compressive strength. Similarly, Zhang et al. [33] proposed an SSA-BP neural network model for predicting the strength of slag–cement stabilized soil. The results showed a 53.4% reduction in prediction error compared with traditional BP neural networks, along with improved convergence speed and generalization ability. Their study identified mineral powder content as the key variable influencing clay strength. Li et al. [34] used a random forest model to predict the compressive strength of basalt fiber reinforced concrete, achieving an 8% and 18.8% lower mean squared error than BP neural networks and support vector regression, respectively. In another study, Chen et al. [35] applied an LSTM model to predict the compressive strength of high-strength concrete, attaining an R^2^ of 0.997, RMSE of 0.508, and MAE of 0.08, all of which surpassed the performance of an SVR model. SHAP analysis in their work confirmed the water–cement ratio as the main factor governing strength. Based on the above studies, it can be concluded that material strength is complexly influenced by multiple factors, including raw material properties, mix gradation composition, curing conditions, and microstructural evolution. Moreover, different prediction models show considerable variation in accuracy, robustness, computational efficiency, and interpretability, and their applicability often depends on the specific material system and engineering context.

Based on the research described above, this paper systematically investigated the influence of steel slag content on the mechanical properties of the mixture and revealed the mechanism of the mechanical properties’ enhancement by cement-stabilized steel slag through SEM-EDS and XRD microstructural characterization techniques. Based on mechanical property data, a CNN-GRU-Attention prediction model was constructed and compared with the CNN-LSTM-Attention, CNN-BiLSTM-Attention, LSTM, and GRU-LSTM models to systematically evaluate the prediction accuracy, convergence speed, and generalization ability of each model. Furthermore, the SHAP explainable analysis method was introduced to explore the key factors influencing the strength of cement-stabilized steel slag, their mechanisms of action, and to quantify the contribution of different input variables. This research aims to provide theoretical reference and technical support for the optimization of the mix proportion and accurate strength prediction of cement-stabilized steel slag materials.

## 2. Materials and Methods

### 2.1. Raw Materials

**Figure 1:**
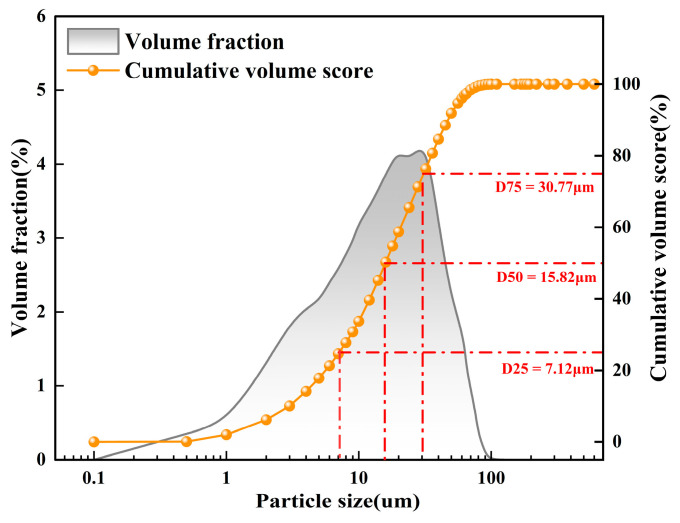
Particle size distribution of cement.

The P·O 42.5 cement used in this study was produced by Xinjiang Tianshan Cement Co., Ltd., Urumqi, China. Its chemical composition and physical properties are shown in Table 1. Particle size distribution was measured using a Dandong Baite BT-9300SE laser particle size analyzer (Dandong Baite Instrument Co., Ltd., Dandong, China), and the results are presented in Figure 1.

**Figure 2:**
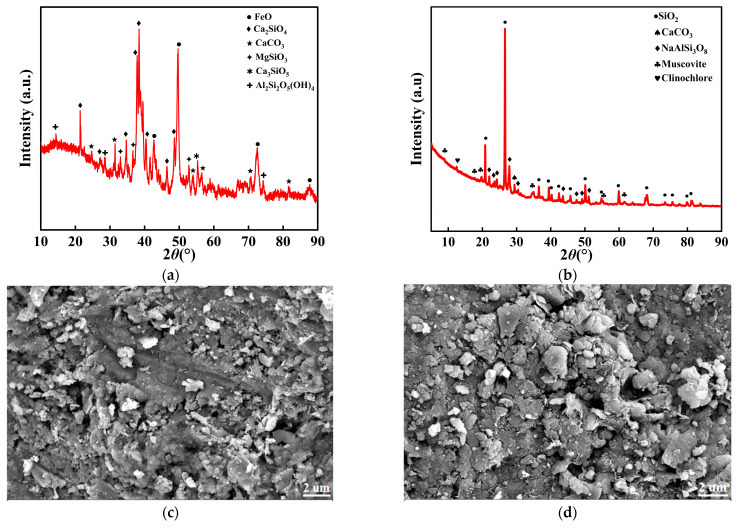
Raw material inspection. (**a**) X-ray diffraction pattern of SSA. (**b**) X-ray diffraction pattern of GG. (**c**) The microstructure of SSA. (**d**) The microstructure of GG.

The steel slag aggregate (SSA) used in this study was converter slag supplied by the Xinjiang Bayi Iron and Steel Factory (Baosteel Group Xinjiang Bayi Iron and Steel Co., Ltd., Urumqi, China). Its key properties are listed in Table 2. The graded gravel (GG) aggregate was obtained from a quarry in the Toudunhe District, Urumqi, China. The phase composition and microstructure of the steel slag and the GG were examined using X-ray diffraction (XRD, Rigaku SmartLab, Rigaku, Tokyo, Japan) and scanning electron microscopy (SEM, ZEISS Sigma 300, ZEISS, Oberkochen, Germany), respectively. The XRD pattern and SEM image of the steel slag are shown in Figure 2a,c [2]; the XRD patterns and microstructures of the GG are presented in Figure 2b,d.

### 2.2. Mix Design

**Figure 3:**
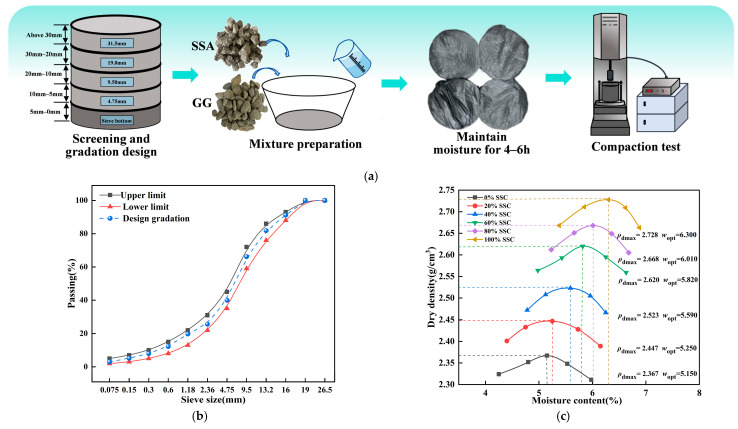
Mix design. (**a**) Mix design process. (**b**) Gradation design results. (**c**) Compaction test results.

The mix design procedure is shown in Figure 3a. Through sieve analysis, both steel slag aggregate (SSA) and graded gravel (GG) were classified into three fractions: 0–5 mm, 5–10 mm, and 10–20 mm, with the gradation composition determined for each. The gradation design followed the C-B-2 specification for cement-stabilized macadam as per the Technical Specifications for Construction of Highway Pavement Base Courses (JTG/T F20-2015) [36]. In this design, SSA was used as a volume-based replacement for GG. Six steel slag content (SSC) levels were tested: 0%, 20%, 40%, 60%, 80%, and 100%, with the cement content fixed at 3.5%. Heavy compaction tests were performed to determine the maximum dry density and optimum moisture content; the results are presented in Figure 3b,c [2].

### 2.3. Mechanical Properties Testing

**Figure 4:**
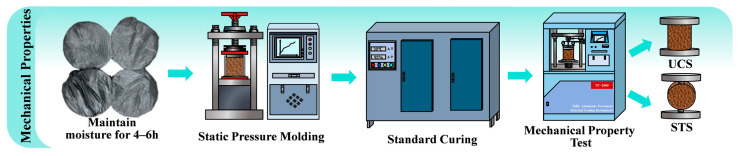
Experimental procedure for mechanical properties.

Mechanical properties tests were conducted in accordance with Test Method T0843-2009 from the Specification for Testing Inorganic Binder Stabilized Materials for Highway Engineering (JTG 3441-2024) [37]. Cylindrical specimens (150 mm × 150 mm) were prepared with a compaction degree of 98%, demolded, and subsequently placed in a standard curing chamber for standard curing. Following the procedures specified in T0805-2024 [37] and T0806-1994 [37], the unconfined compressive strength (UCS) and splitting tensile strength (STS) of the mixtures were determined at curing ages of 7, 28, 60, and 90 d. A schematic of the experimental procedure is shown in Figure 4.

### 2.4. Micro-Scale Testing Methods

**Figure 5:**
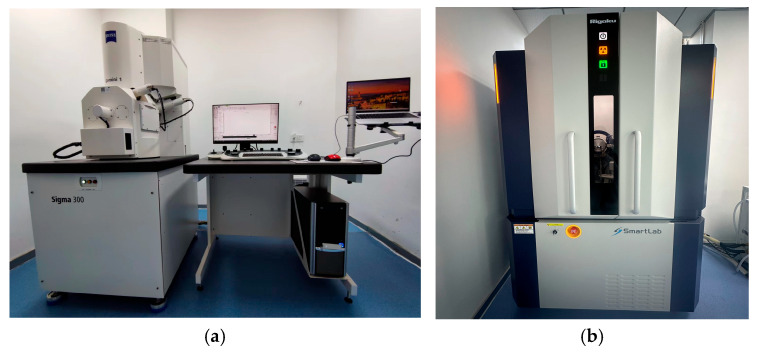
Microscopic testing equipment. (**a**) Scanning electron microscopy. (**b**) X-ray diffraction.

Scanning Electron Microscopy (SEM) Testing: Microstructural characterization of the samples after different curing ages was performed using a ZEISS Sigma 300 field emission scanning electron microscope (FE-SEM, ZEISS, Oberkochen, Germany). For sample preparation, particles with a maximum diameter of 5 mm were selected and mounted on a copper stage with conductive adhesive tape. The samples were then sputter-coated with gold under high vacuum to enhance conductivity before high-resolution SEM observation. The equipment setup is shown in Figure 5a.

X-ray Diffraction (XRD) Testing: The phase composition of the mixture samples cured for different durations was characterized using a Rigaku SmartLab X-ray diffractometer (Rigaku Corporation, Akishima, Tokyo, Japan). Sample preparation involved drying the samples to constant weight in an oven, followed by grinding to a particle size below 75 μm. The resulting powder was evenly spread on the sample holder for scanning over a 2θ range of 5–90° at a rate of 2°/min. The X-ray diffractometer used in this test is presented in Figure 5b.

### 2.5. Database Construction

This study integrated experimental data with data from the literature to construct a comprehensive dataset containing 168 entries for UCS and 126 entries for STS. The UCS dataset includes 24 sets of measurements obtained in this study, supplemented by 144 sets from previously published literature [2]. Similarly, the STS dataset comprises 18 sets from the present experiments and 108 sets from the same literature source. All data are well-defined, with complete input and output parameters. The dataset was partitioned such that 80% of the data formed the training set, with 15% of this training subset held out as a validation set; the remaining 20% served as the test set. The steel slag content (SSC), cement content (CC), gradation type (GT), compaction degree (CD), and curing period (CP) were selected as input parameters, while the USC and STS of the mixture were used as output parameters.

### 2.6. Establishment of the Prediction Model

**Figure 6:**
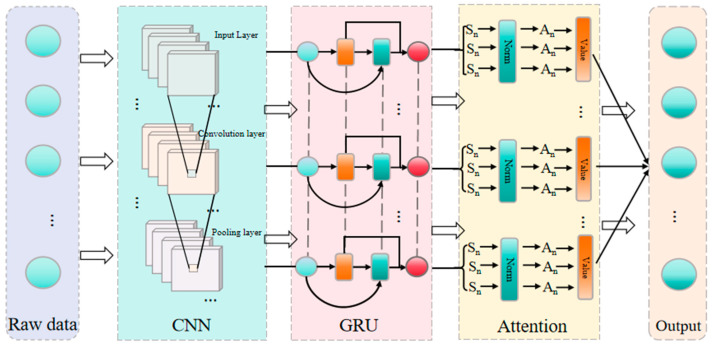
Prediction flow of the CNN-GRU-Attention Model.

This paper integrated convolutional neural networks (CNNs), gated recurrent units (GRUs), and attention mechanisms to construct a CNN-GRU-Attention hybrid prediction model, the framework of which is illustrated in Figure 6. Within this architecture, the CNN component serves as a feature extractor. It employs two 2D convolutional layers with 32 and 64 1 × 1 kernels, respectively, to identify deep local patterns and nonlinear relationships within the multi-dimensional input variables. The extracted features are subsequently fed into a GRU layer for modeling dependencies and capturing complex nonlinear interactions among the parameters. Finally, an Attention module dynamically allocates weights to different feature channels, focusing computational resources on the most informative features while suppressing irrelevant noise, thereby enhancing prediction accuracy.

For comparative analysis, four additional models (CNN-LSTM-Attention, CNN-BiLSTM-Attention, LSTM, and GRU-LSTM) were constructed. To ensure a fair comparison, all models underwent an identical hyperparameter optimization process to determine the optimal learning rate and number of hidden units. To prevent overfitting, an early stopping mechanism was employed, which automatically terminated training if the validation loss showed no significant improvement over 50 consecutive epochs. Furthermore, to rigorously evaluate the generalization capability and statistical stability of the proposed architecture, a 5-fold cross-validation strategy repeated 5 times was implemented during the evaluation phase. This approach ensures that the reported performance metrics are robust and independent of any specific random data split, providing a comprehensive assessment across the entire experimental database. Finally, to enhance the interpretability of the model’s decisions, the SHAP (Shapley Additive exPlanations) method was introduced for post hoc explanation of the optimal model. By calculating the Shapley values for test samples, this method quantifies the marginal contribution of each input feature, clearly revealing the key influencing factors and their directional effects on the mechanical properties.

The performance of the models was evaluated using three quantitative metrics: Mean Absolute Error (MAE), Root Mean Square Error (RMSE), and the Coefficient of Determination (R^2^). The computational procedures for these metrics are provided in Equations (1)–(3). MAE directly reflects the average absolute deviation between the predicted and actual values. Expressed in the same units as the target variable, it provides an intuitive measure of the typical accuracy of the model and is relatively insensitive to outliers. A value closer to zero indicates a higher model accuracy. RMSE amplifies the contribution of larger errors through squaring, making it more sensitive to significant deviations in predictions. Its magnitude reveals whether the model exhibits severe individual prediction errors, serving as a key indicator for assessing prediction stability. A value closer to zero corresponds to higher model precision. R^2^ quantifies the proportion of variance in the target variable that is explained by the model, measuring its ability to capture the overall variation trend in the data. A value closer to 1 signifies a stronger explanatory power of the model. If a model exhibits a high R^2^ but large MAE and RMSE values, it may indicate the presence of systematic bias. Conversely, if the MAE and RMSE are low while R^2^ remains modest, it suggests that the model may not adequately capture the underlying patterns in the data. These three metrics validate the model from three distinct dimensions: absolute error, dispersion, and correlation. Their combined use helps mitigate the limitations associated with relying on a single indicator.

(1) $$MAE=\frac{1}{n}\sum_{i=1}^{n}\left|y^{obs}-y^{pred}\right|$$

(2) $$RMSE=\sqrt{\frac{\sum_{i=1}^{n}{\left(y^{obs}-y^{pred}\right)}^{2}}{n}}$$

(3) $$R^{2}=1-\frac{\sum_{i=1}^{n}{\left(y^{obs}-y^{pred}\right)}^{2}}{\sum_{i=1}^{n}{\left(y^{obs}-y^{\bar{obs}}\right)}^{2}}$$

In the formula, $y^{\mathrm{obs}}$$y^{pred}$ denotes the observed value and the predicted value.

## 3. Results and Discussion

### 3.1. Results of Mechanical Properties Testing

**Figure 7:**
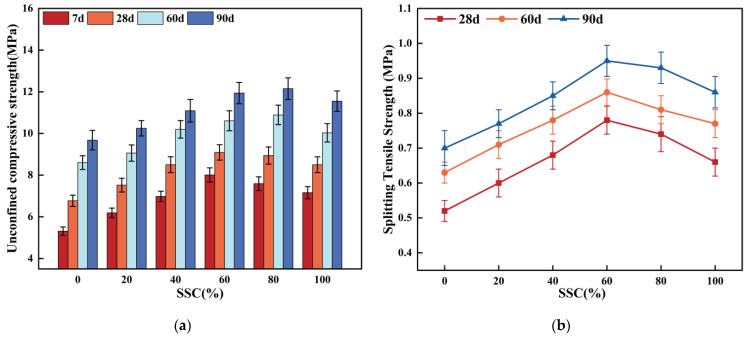
Experimental results of mechanical properties. (**a**) UCS test results. (**b**) STS test results.

Based on the compaction test results, mechanical property tests were performed to determine the UCS and STS of CSSS under different curing ages and SSC levels, as shown in Figure 7. Both UCS and STS displayed marked nonlinear evolution over time. After 7 d of curing, the 60% SSC group developed a robust skeleton with the aggregates due to the coarse, porous surface of the steel slag. Through physical filling and mechanical interlocking, its UCS reached 8.01 MPa, representing a 50.8% increase over the control group. At this early stage, when hydration was not yet vigorous and inert components in the steel slag remained inactive, strength mainly depended on cement hydration and aggregate interlocking effect. The 60% SSC group showed more uniform paste distribution, leading to better early performance than the 80% SSC group. By 90 days, however, the 80% SSC group achieved a UCS of 12.15 MPa, a 25.5% increase over the control group and slightly higher than the 60% SSC group (11.94 MPa). This is attributed to sustained cement hydration, which produced Ca(OH)_2_ and established a moderately alkaline environment. This gradually eroded the protective film on the steel slag surface, activating latent hydration activity and inducing secondary reactions that generated substantial C-S-H gel and CaCO_3_, filling micropores. Such micro-reinforcement from chemical activity became more pronounced at higher SSC, resulting in significant late-strength gains. STS indirectly reflects the tensile strength of the material, and its trend was similar to that of USC, showing an initial increase followed by a decrease, but its peak values in both the early and later stages were 60% of SSC. After 90 d of curing, the STS of the 60% SSC group reached 0.95 MPa, representing a 35.7% increase compared to the control group. After 90 days of curing, the STS of the 60% SSC group reached 0.95 MPa, representing a 35.7% increase compared to the control group. This gain in efficiency was significantly better than that observed for UCS. This difference arises because UCS depends mainly on the physical interlocking framework between aggregates, whereas STS is highly sensitive to bond strength within the interfacial transition zone (ITZ). At a 60% steel slag content, the interlocking effect formed between the porous and rough surface characteristics of the steel slag and the cement paste reached its optimal state, significantly enhancing the interfacial tensile strength. When the steel slag content exceeds the optimal threshold, the inherent porosity of the steel slag leads to an increase in harmful pores in the steel slag–cement composite paste, resulting in excessive overall porosity of the mixture. Furthermore, the slow hydration reaction of its potential cementing minerals, C_2_S and C_3_S, makes it difficult to effectively compensate for the strength loss caused by increased porosity in the early stages. The negative effects of increased steel slag content on strength development outweigh the positive contributions, resulting in a decrease in overall strength.

### 3.2. Microstructural Analysis

**Figure 8:**
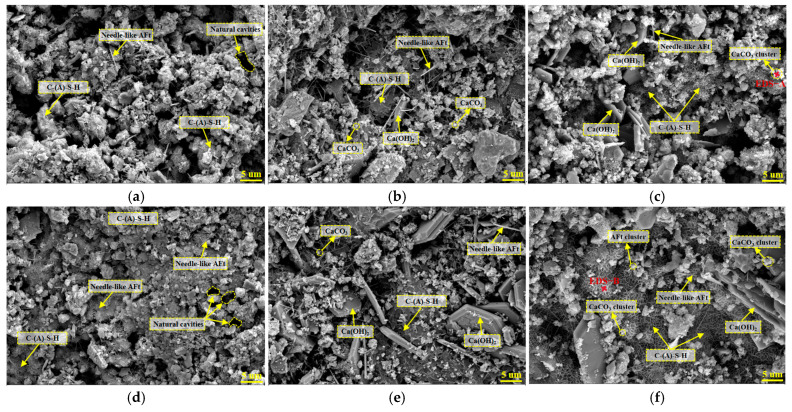
Microstructural morphology at different stages of development. (**a**) 28 d-0%SSC; (**b**) 60 d-0%SSC; (**c**) 90 d-0%SSC; (**d**) 28 d-60%SSC; (**e**) 60 d-60%SSC; (**f**) 90 d-60%SSC.

**Figure 9:**
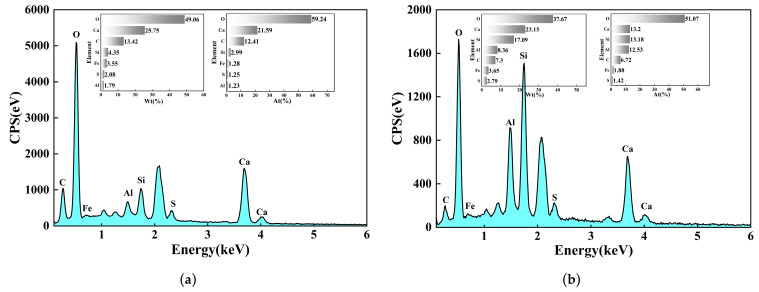
EDS Analysis. (**a**) EDS spectrum at point A (CaCO_3_). (**b**) EDS spectrum at point B (C-S-H).

Figure 8 compares the microstructural evolution patterns of the control group (0% SSC) and the experimental group (60% SSC) at different ages. At 28 d (Figure 8a,d), hydration products, including C-(A)-S-H gel, were observed in both groups. Compared with the control, the microstructure of the experimental group appeared more consolidated, with a seemingly higher frequency of ettringite (AFt) crystals. This observation was attributed to the incorporation of steel slag. The dissolution of steel slag releases Ca^2+^ ions into the pore solution, elevating the local Ca^2+^ concentration. According to the principle of chemical equilibrium, this shift can promote the precipitation of AFt by facilitating its reaction with aluminate and sulfate ions (primarily from the cement). The formed AFt crystals were observed within the matrix, suggesting that this micro-filling effect contributed to the initial enhancement of the structural compactness in the experimental group. At 60 days (Figure 8b,e), as hydration progressed, both groups developed interconnected C-(A)-S-H gel networks. A notable difference was the more prevalent presence of hexagonal-platelet calcium hydroxide (CH) crystals in the experimental group. This is primarily ascribed to the continued hydration of free CaO (f-CaO) from the steel slag. It is inferred that the formation of CH crystals, along with their potential subsequent carbonation, contributed to pore filling and microstructural densification. The combined solid-volume increase from these processes is consistent with a refined pore structure. It is noted that although the delayed hydration of f-CaO poses a potential risk of long-term expansion, this risk is effectively managed by using aged steel slag (over one year) with a limited f-CaO content (below 2%) and a controlled replacement ratio. After 90 d of curing (Figure 8c,f), combined with EDS point analysis (Figure 9a), spherical features identified as calcium carbonate (CaCO_3_) were observed in association with CH crystals. This is interpreted as the result of natural carbonation of CH. Such deposition is expected to refine the pore structure and promote further densification within the cementitious system of the experimental group. Meanwhile, the EDS spectrum in Figure 9b confirmed that the reticular gel-like product contained Ca, Al, and Si, which is indicative of C-A-S-H formation. This indicates that reactive components in the steel slag continued to participate in pozzolanic reactions over time. These observed microstructural features align with the sustained improvement in the macroscopic mechanical properties of the composite.

### 3.3. X-Ray Diffraction Analysis

**Figure 10:**
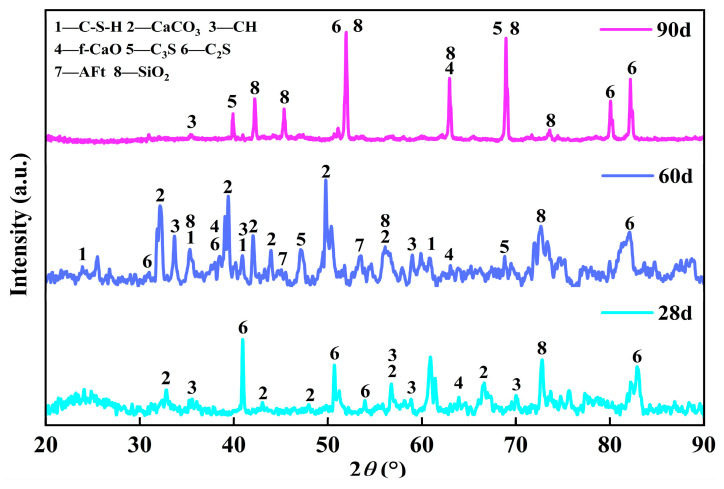
XRD patterns at different stages of development.

X-ray diffraction analysis was performed on the mixture containing 60% steel slag at curing ages of 28, 60, and 90 d, with the results presented in Figure 10. As shown in the figures, the evolution of the XRD patterns indicates a transition from a predominantly amorphous state towards a more crystalline system with increasing curing age. After 28 d of curing, the XRD pattern was characterized by a pronounced diffuse background and broadened diffraction peaks. This is indicative of a system where the primary cementitious phase is a poorly crystalline, long-range-disordered C-(A)-S-H gel. This observation aligns with the early-stage microstructure observed in Figure 8d, where hydration products were not yet extensively developed. After 60 d of curing, a noticeable increase in the intensity of characteristic diffraction peaks corresponding to CaCO_3_ was observed. This suggests that CH crystals, resulting from the hydration of free CaO (f-CaO) in the steel slag, underwent carbonation, forming calcium carbonate crystals that contributed to microstructural filling. This phase evolution is consistent with the morphological changes observed in Figure 8e. After 90 days of curing, the XRD patterns revealed distinct diffraction peaks for crystalline phases such as C_2_S and SiO_2_. This implies the persistence of unreacted steel slag particles, confirming their slow hydration kinetics. These particles, composed of high-hardness crystalline minerals, are considered to act as more than conventional micro-fillers. It is proposed that they function as incompressible rigid skeletons within the matrix, sharing and transferring stress, which may inhibit the initiation and propagation of microcracks. Furthermore, their crystalline surfaces could provide substrates for the deposition of C–S–H and other gel products, potentially guiding the ordered growth of these phases and contributing to the dense microstructure shown in Figure 8f. Therefore, the observed sustained increase in later-age strength can be rationalized by a synergistic “rigid skeleton–gel densification” mechanism, which is inferred to enable continuous strengthening of the matrix and improvement in macroscopic performance.

## 4. Analysis of Model Prediction Results

### 4.1. Results of a Single Data Split

**Figure 11:**
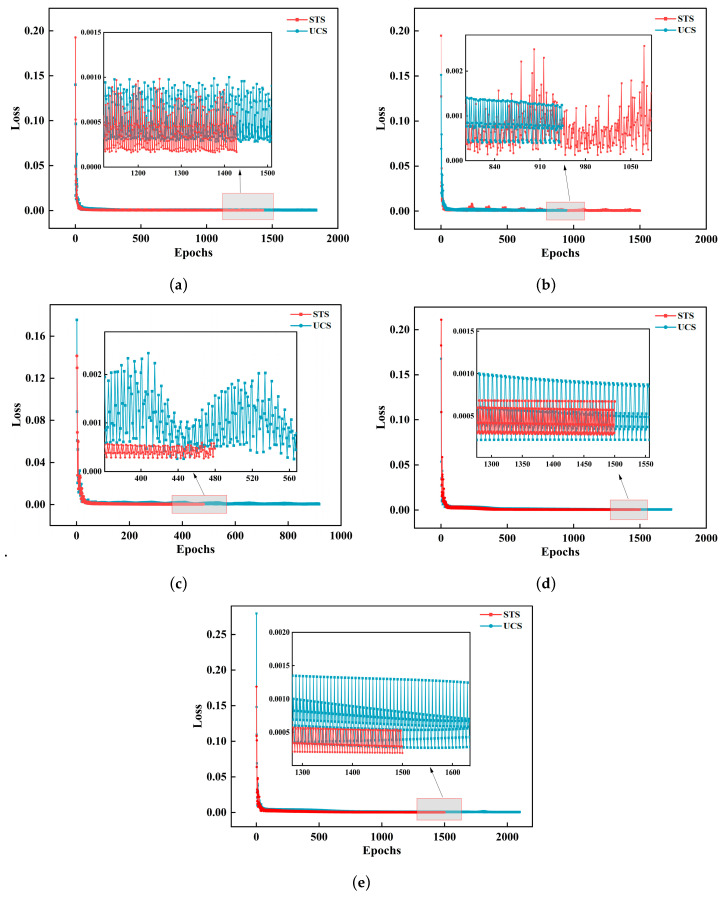
Comparison of model iteration loss. (**a**) CNN-GRU-Attention model loss. (**b**) CNN-BiLSTM-Attention model loss. (**c**) CNN-LSTM-Attention model loss. (**d**) GRU-LSTM model loss. (**e**) LSTM model loss.

To evaluate the convergence performance of the CNN-GRU-Attention model, its iterative loss was compared with that of the CNN-LSTM-Attention, CNN-BiLSTM-Attention, LSTM, and GRU-LSTM models, as shown in Figure 11. All models exhibited good convergence trends: the loss value decreased rapidly from the range of 0.15–0.20 to below 0.02 within 100–200 training iterations, and then entered a stable phase. During the stable phase, the CNN-BiLSTM-Attention, CNN-LSTM-Attention, GRU-LSTM, and LSTM models all showed some fluctuations in their STS and UCS predictions. The loss curve of CNN-GRU-Attention was smoother and did not show significant oscillations, indicating that its training process had higher robustness and numerical stability. In terms of convergence speed, the CNN-LSTM-Attention model trained the fastest, with its UCS prediction stabilizing after approximately 900 iterations; the CNN-BiLSTM-Attention, CNN-GRU-Attention, and LSTM models required approximately 1500–1800 iterations; and the GRU-LSTM model converged the slowest, stopping after about 2000 iterations. In terms of convergence, the loss values of all models decreased to a low level. Comparing the prediction losses of the two types of intensity indicators, it can be seen that the steady-state loss of STS was generally lower than that of UCS. The STS loss fluctuated within the range of 0.0005 to 0.0010, while the UCS loss was concentrated in the range of 0.0010 to 0.0020. This difference mainly stems from the different numerical magnitudes of the two types of metrics; the average absolute value of SS was approximately 1/10 of that of UCS, resulting in smaller fluctuations during gradient updates and making the model relatively less sensitive to STS loss. In terms of fluctuation amplitude during the steady-state phase, CNN-GRU-Attention exhibited the most stable performance, with its UCS and STS losses remaining within the range of 0.0002–0.0010, and the STS loss further concentrated between 0.0002 and 0.0005. In contrast, CNN-BiLSTM-Attention and CNN-LSTM-Attention showed larger fluctuations in loss during STS or UCS prediction. Both GRU-LSTM and LSTM exhibited noticeable “sawtooth” oscillations during the later stages of convergence, indicating that the parameters repeatedly fluctuated around local minima, resulting in relatively poor prediction stability. Overall, CNN-GRU-Attention showed smoother convergence during training, suggesting that this model possesses superior convergence quality when handling such complex material property prediction tasks.

**Figure 12:**
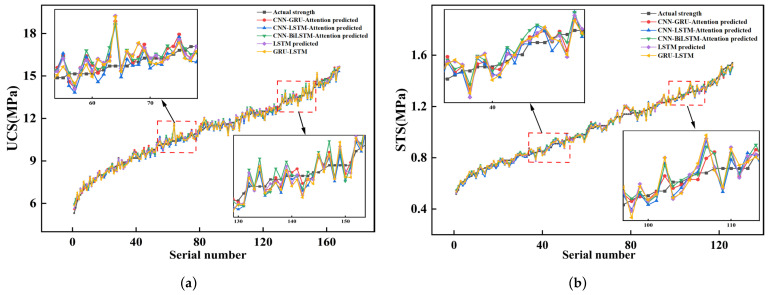
Comparison of model prediction results. (**a**) Comparison of UCS prediction results across models. (**b**) Comparison of STS prediction results across models.

To validate the predictive capability of the models, experimental measurement data were compared with the prediction data from the CNN-GRU-Attention, CNN-LSTM-Attention, CNN-BiLSTM-Attention, LSTM, and GRU-LSTM models, as shown in Figure 12. Figure 12 showed that all models effectively captured the overall trend of strength development in both UCS and STS. Although the predicted strengths fluctuated around the measured data, the CNN-GRU-Attention model aligned significantly more closely with the experimental values than the other models, demonstrating superior dynamic tracking ability. Local enlargements in Figure 12 revealed that in regions where strength varied sharply due to changes in mix proportions, the experimental data exhibited notable fluctuations. Models such as LSTM and GRU-LSTM often displayed prediction lags or amplitude deviations in these areas. In contrast, the CNN-GRU-Attention model showed the smallest deviation between the predicted and measured values, highlighting its stronger ability to track dynamic changes and reduce prediction delay. This performance advantage stems from the model’s architecture: the CNN component automatically identifies and amplifies feature combinations that are critical to mechanical properties, effectively extracting deep spatial correlations among input variables such as CC and SSC. This process supplies higher-quality feature vectors for subsequent computation. The GRU structure, being more streamlined than LSTM, enables faster convergence to a global optimum within the sample size of this study while preserving the ability to model nonlinear relationships. Furthermore, the attention mechanism allows the model to dynamically weigh different input features, effectively “focusing” on the core factors influencing strength.

The predictive evaluation metrics for the five models in forecasting UCS and STS are presented in Table 3. The table indicates that all five models demonstrated high predictive accuracy, with R^2^ values exceeding 0.96 for both the test and training datasets. Among these, the CNN-GRU-Attention model exhibited optimal predictive performance across all evaluation metrics. On the training set, the R^2^ values for each model ranged between 0.9824 and 0.9910, indicating that all architectures effectively established nonlinear mappings between input features and mechanical responses. Regarding the training set, the CNN-GRU-Attention model demonstrated superior predictive capability, achieving training set R^2^ values of 0.9856 and 0.9910 for UCS and STS predictions, respectively, while also yielding the lowest RMSE and MAE values among comparable models. For the test set, the CNN-GRU-Attention model achieved an R^2^ of 0.9904 for UCS prediction, surpassing the baseline LSTM model’s result of 0.988. Its RMSE of 0.2380 MPa and MAE of 0.2078 MPa were the lowest among all models, with no significant fluctuation in error compared to the training set, which indicated no overfitting. For STS prediction, although the test set accuracy of each model was slightly lower than that for UCS, the model achieved an R^2^ of 0.9863, with an RMSE of 0.0240 MPa and an MAE of 0.0198 MPa, significantly outperforming other models. A comparison across the entire dataset revealed that the CNN-GRU-Attention model reduced the overall RMSE by 11.63% and MAE by 6.32% compared to the traditional LSTM model. Against the more complex CNN-BiLSTM-Attention model, its RMSE and MAE decreased by 11.96% and 8.89%, respectively. Performance improvements were particularly pronounced in STS forecasting with smaller data samples. The CNN-GRU-Attention model achieved a maximum reduction of 25.00% in overall RMSE compared to the CNN-BiLSTM-Attention model, and MAE reduction rates relative to other models ranged between 13.01% and 26.69%.

**Figure 13:**
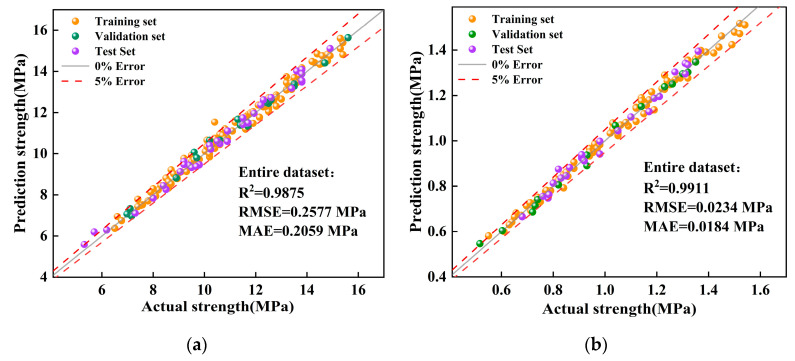
Scatter plot of prediction results from the CNN-GRU-Attention model. (**a**) UCS prediction results. (**b**) STS prediction results.

The scatter plot of predicted versus actual values for the CNN-GRU-Attention model is presented in Figure 13. The model demonstrated high prediction accuracy for both UCS and STS. Overall, the data distribution across the training, validation, and test sets was balanced over the entire strength range, effectively covering both high and low intervals, which minimized the influence of data distribution bias on prediction results. From the graphical distribution, the data points in the training, validation, and test sets were highly concentrated around the y = x line, and the vast majority of sample points fell within the relative error band of y = x(1 ± 5%), indicating a low residual level. Although these results from a single hold-out split suggest strong predictive performance, one partition alone cannot definitively rule out overfitting or quantify the model’s sensitivity to data composition. Therefore, repeated k-fold cross-validation was conducted to provide a statistically robust evaluation of model stability and generalization capacity.

### 4.2. Predictive Results of Cross-Validation

To eliminate potential bias from a single random data split and to rigorously assess the model’s generalization capability, a 5-fold cross-validation strategy was implemented, repeated 5 times. This process involved randomly partitioning the total dataset into five disjoint subsets; in each of the 25 independent runs (5 folds × 5 repetitions), four subsets were utilized for training while the remaining one was used for performance testing. This repeated-validation approach accounts for stochastic variability in the data distribution and ensures that the model’s performance is statistically significant. The statistical results, including the mean values and standard deviations of R^2^, RMSE, and MAE across the 25 cycles, are summarized in Table 4.

As shown in Table 4, the CNN-GRU-Attention model achieved the highest average R^2^ values for UCS and STS predictions (0.9417 and 0.9329, respectively) while also attaining the lowest RMSE and MAE among the compared architectures. In terms of robustness, the standard deviation of R^2^ for STS predictions was 0.024 for the proposed model, which was lower than the 0.036 observed for the conventional GRU-LSTM model. Notably, for STS prediction, all three CNN hybrid models incorporating the Attention mechanism achieved comparable mean absolute error values (approximately 0.050) and exhibited a similarly narrow range of standard deviations. This consistency suggests that the Attention mechanism plays a stabilizing role in controlling prediction bias, aiding in the reduction in error distribution variance across different model variants. The error metrics of the proposed model also exhibited relatively small variations across multiple experimental runs, indicating that the model maintains a stable predictive performance across different data partitions. This sustained performance suggests that the model effectively mitigates the risk of overfitting to specific data noise. By integrating CNN-based feature extraction with Attention-driven weight allocation, the model is able to learn the generalized nonlinear relationships within the studied mix proportions. This stability is expected to enhance prediction consistency when confronted with similar material fluctuations, thereby providing a reliable tool for the mechanical performance evaluation of CSSS materials. As can be seen from Table 3 and Table 4, there was a difference between the average performance obtained through cross-validation and the single-split results. This indicates that a single split may be influenced by specific data partitioning, while the 5-fold cross-validation evaluates the average performance of the model on different data subsets through repeated sampling. The small standard deviation further demonstrates the high stability of the CNN-GRU-Attention architecture.

### 4.3. SHAP Feature Attribution and Correlation Analysis

**Figure 14:**
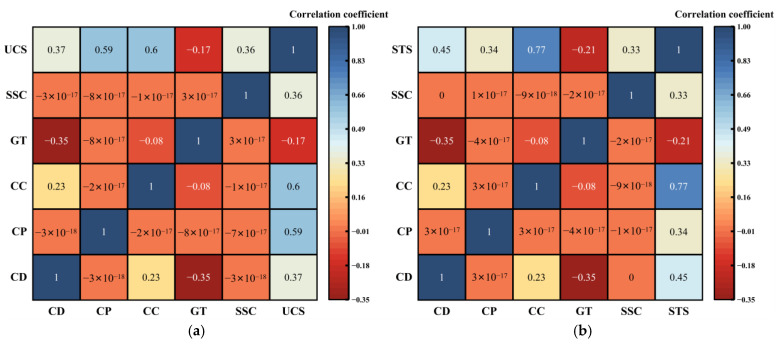
Correlation matrix between characteristic variables and mechanical properties. (**a**) Feature variable correlation matrix with UCS intensity. (**b**) Feature variable and STS intensity correlation matrix.

The results presented above indicate that the CNN-GRU-Attention model possesses high predictive accuracy, strong generalization capability, and stable convergence. To further address the “black-box” limitation of deep learning models and clarify the intrinsic physical mechanisms underlying the evolution of mechanical properties in CSSS, this study employed Pearson correlation analysis and SHAP interpretability theory to examine the decision-making process of the CNN-GRU-Attention model in depth. The Pearson correlation coefficients between each input feature and the mechanical performance indicators are shown in Figure 14.

Analysis of Figure 14 revealed that for both UCS and STS, CC exhibited a strong positive correlation with mechanical properties. The correlation coefficient between CC and UCS was 0.60, and between CC and STS was 0.77. Mechanistically, this is attributed to the cementitious components such as C-S-H formed during cement hydration, which bind steel slag and aggregate particles and constitute the primary source of strength development. In contrast, STS demonstrated higher sensitivity to CC, as STS reflects the material’s crack resistance under tensile stress and depends significantly on the bond quality within the ITZ between the cementitious phase and aggregates. Increasing CC directly enhances the thickness and continuity of the cementitious phase at the interface, thereby improving the mechanical behavior of the ITZ and exerting a more direct influence on STS. UCS, however, is influenced to a greater extent by physical interlocking between particles and the overall skeletal structure, making it relatively less sensitive to CC variations than STS. Both CD and CP also showed strong positive contributions to strength. In UCS prediction, CP had a correlation coefficient of 0.59 and CD 0.37, consistent with typical strength development patterns of cement-stabilized materials. For STS prediction, the contribution of CD increased to 0.45, indicating that appropriate compaction significantly enhances intergranular interlocking and supports the evolution of indirect flexural strength. SSC exhibited a moderately positive correlation with strength, with coefficients of 0.36 for UCS and 0.33 for STS, suggesting that steel slag contributes to overall strength through its inherent mechanical properties and potential hydration activity, though its effect remains secondary to that of cementitious binding. Conversely, GT showed negative correlations with both UCS and STS, implying that variations in GT may compromise the internal skeleton stability and thereby weaken strength. Furthermore, correlation coefficients between the different input features were generally close to zero, confirming the high independence among variables in the experimental design and effectively mitigating multicollinearity during model training.

**Figure 15:**
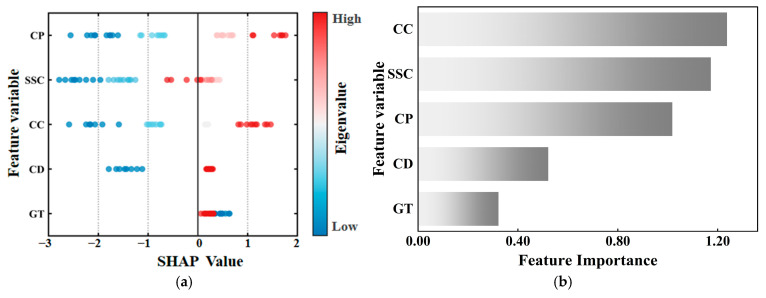

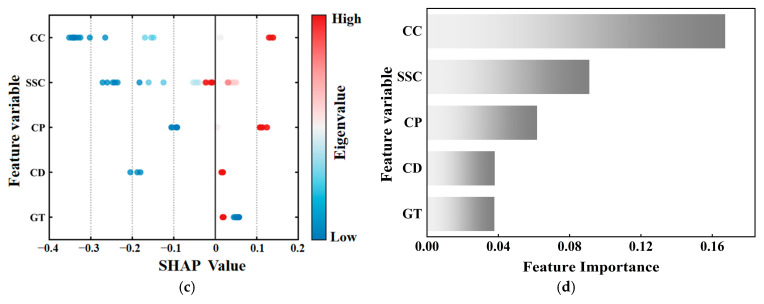
SHAP analysis of mechanical properties. (**a**) SHAP Feature contributions for UCS. (**b**) SHAP Feature importance for UCS. (**c**) SHAP Feature contributions for STS. (**d**) SHAP Feature importance for STS.

The SHAP importance analysis is shown in Figure 15. The figure shows that both CC and CP play a dominant role in predicting UCS and STS. Among them, CC had the highest average absolute SHAP value, with high values concentrated in the positive SHAP range and low values distributed in the negative range, further confirming that CC was the most critical factor affecting material strength. Besides, CP showed a consistently positive correlation in both types of strength prediction. This statistical trend was explicitly corroborated by our microstructural observations at different curing ages. As the curing age increased, the SEM images revealed that hydration products such as C-S-H gels continuously nucleated and grew, effectively filling the internal capillary pores and densifying the matrix. This microstructural refinement explains the sustained positive SHAP contribution of CP to macroscopic strength. In the STS prediction model, lower steel slag content showed a negative impact. This indicates that there is a threshold effect in the strengthening effect of steel slag content on strength. The SHAP-identified importance of steel slag content is closely linked to its contribution to microstructural density. As indicated by the preceding SEM analysis, the steel slag-modified samples showed that an appropriate increase in steel slag content promotes the generation of more hydration products, which fill inter-particle voids and result in a more compact matrix structure. Appropriate addition can improve strength through physical support and active effects, but excessive addition will cause strength fluctuations due to changes in the void structure and moisture content of the mixture. The contribution of CD to STS is relatively concentrated, indicating that although its influence is stable, its weight is lower than the cementing effect of cement. High GT values generally correspond to negative SHAP values, while low values correspond to positive values, suggesting that using the C-B-2 type gradation with smaller overall particle sizes is detrimental to the formation of aggregate interlock, reducing the density and stability of the aggregate skeleton, and thus negatively impacting strength development.

Integrating the results from both the Pearson correlation and SHAP analysis, the factors influencing the strength of CSSS can be ranked as follows: CC > SSC > CP > CD > GT. Therefore, in practical mix design, CC and SSC should be prioritized as the primary design parameters, and GT should be optimized.

## 5. Conclusions

This study proposes an integrated framework that combines microstructural evolution, macroscopic performance prediction, and intelligent design feedback to optimize material properties. The framework integrates multi-scale characterization, deep learning, and SHAP-based interpretability analysis to reveal the underlying mechanisms governing the long-term strength evolution of cement–steel slag systems. The main conclusions are as follows:

(1) Experimental results indicate that the UCS and STS of cement-stabilized steel slag exhibit significant nonlinear growth with age. When the curing period was 7 days, 60% SAA, due to its rough and porous surface, which formed a stable physical interlock with the aggregates, had a UCS of 8.01 MPa, which was 50.8% higher than that of the control group. After curing for 90 d, the activity of 80% steel slag was activated in the alkaline environment provided by continuous hydration. The C-S-H gel and CaCO_3_ generated by secondary hydration effectively filled the pores, increasing the UCS to 12.15 MPa. Meanwhile, the STS of the 60% SSC sample was 0.95 MPa, an increase of 35.7% compared to the control group, demonstrating that an appropriate amount of steel slag can optimize interfacial interlocking and thus improve mechanical properties.
(2) SEM-EDS and XRD analysis of CSSS indicate that the incorporation of steel slag significantly promotes early-stage AFt formation and the development of the C-A-S-H gel network in the intermediate to late stages. With increasing curing age, the cementitious system progressively transforms from an amorphous cementitious state into a highly crystalline structure. Within this process, CH, formed from the hydration of f-CaO in the steel slag, undergoes carbonation, producing CaCO_3_ clusters that effectively fill micro-pores. In the later curing stages, the secondary hydration of active components in steel slag continuously generates C-(A)-S-H gel, working in concert with the rigid framework formed by unreacted C_2_S and SiO_2_ particles. The gel fills interparticle gaps via heterogeneous nucleation, while the hard particles provide physical support. This deep coupling of physical filling and chemical gelation constitutes the physical essence of the sustained strength development in CSSS.
(3) The CNN-GRU-Attention model demonstrated outstanding performance in predicting the mechanical properties of CSSS. In a single data split, it achieved R^2^ values of 0.9875 for UCS and 0.9911 for STS, with corresponding RMSEs of 0.2380 MPa and 0.0240 MPa, and MAEs of 0.2078 MPa and 0.0198 MPa. The model converged smoothly and efficiently, with STS iteration losses ranging between 0.0002 and 0.0005 and UCS losses between 0.0010 and 0.0020, exhibiting no significant oscillations. Furthermore, under rigorous 5 × 5 repeated cross-validation, a robust test of generalization, the model maintained superior and stable predictive capabilities. It achieved the highest average R^2^ (0.9417 for UCS and 0.9329 for STS) and the lowest error metrics, both accompanied by minimal standard deviations. This confirms its reliability and generalization capacity beyond the scope of a specific data partition.
(4) Through correlation and SHAP analysis, it was found that CC is strongly positively correlated with strength and is the main factor influencing strength development. Its hydration and cementation process is the basis for strength development. The effect of SSC exhibits a threshold effect. Appropriate incorporation can provide physical reinforcement and enhance activity, while excessive amounts can cause strength fluctuations due to changes in gradation and water absorption. GT should avoid gradations with overall small particle sizes, as this weakens skeletal stability and consequently constrains overall strength development.

## 6. Limitations and Future Prospects

Although this study establishes a closed-loop framework for predicting performance and analyzing mechanisms of cement-stabilized steel slag (CSSS), the following limitations—stemming from constraints in experimental conditions and data samples—should be addressed in future work:

(1) Current analysis of the microstructural evolution of CSSS relies mainly on morphological observations and qualitative descriptions, with limited quantification. Future research will incorporate quantitative phase analysis via XRD, TG, mercury intrusion porosimetry (MIP), and complementary techniques to obtain quantitative physical parameters such as phase composition and pore distribution. These parameters will then be integrated as features into predictive models to improve objectivity and physical interpretability.
(2) The training and validation data for the current model were derived solely from experiments conducted in this study, with steel slag samples originating from a limited range of sources and processing methods. The model’s generalizability, therefore, requires further verification. In future work, we will systematically collect steel slag samples from diverse sources and processing routes, along with their corresponding multi-mechanical performance data, to build a more representative standardized dataset. This will provide a reliable foundation for extensive model validation and continuous refinement.

## Figures and Tables

**Table 1:** Physical properties and principal chemical composition of cement.

Materials	Specific Surface Area (kg/m^2^)	Density (g/cm^3^)	Setting Time (min)		Compressive Strength (MPa)		Flexural Strength (MPa)		Chemical Composition (%)				
			Initial Setting	Final Setting	3 d	28 d	3 d	28 d	CaO	MgO	SiO_2_	Al_2_O_3_	Fe_2_O_3_
Cement	355	3.22	133	187	26.8	50.2	5.7	9.1	63.2	1.83	22.2	4.63	2.72

**Table 2:** Physical properties of SSA.

Project	Apparent Relative Density (g/cm^3^)			Relative Density of the Mass (g/cm^3^)			Water Absorption Rate (%)	Crushing Value (%)	Load Ratio (%)	Los Angeles Abrasion Value
Particle size (mm)	0–4.75	4.75–9.5	9.5–19	0–4.75	4.75–9.5	9.5–19				
Test value	3.339	3.348	3.352	3.186	3.192	3.187	1.86	15.8	124.7	13.5
Standard value	≥2.6	≥2.6	≥2.6	-	-	-	≤3.0	≤26	≥120	≤28

**Table 3:** Results of a Single Data Split.

Type	Model	Training Set			Test Set			Overall Data		
		R^2^	RMSE (MPa)	MAE (MPa)	R^2^	RMSE (MPa)	MAE (MPa)	R^2^	RMSE (MPa)	MAE (MPa)
UCS	CNN-GRU-Attention	0.9856	0.2683	0.2106	0.9904	0.2380	0.2078	0.9875	0.2577	0.2059
	CNN-LSTM-Attention	0.9837	0.2858	0.2233	0.9845	0.3022	0.2468	0.9844	0.2878	0.2295
	CNN-BiLSTM-Attention	0.9824	0.2964	0.2257	0.9847	0.2997	0.2399	0.9839	0.2927	0.2260
	LSTM	0.9829	0.2920	0.2146	0.9882	0.2635	0.2169	0.984	0.2916	0.2198
	GRU-LSTM	0.9832	0.2895	0.2156	0.9822	0.3236	0.2767	0.982	0.3031	0.2326
STS	CNN-GRU-Attention	0.9910	0.0239	0.0186	0.9863	0.0240	0.0198	0.9911	0.0234	0.0184
	CNN-LSTM-Attention	0.9850	0.0304	0.0245	0.9702	0.0353	0.0288	0.9842	0.0312	0.0251
	CNN-BiLSTM-Attention	0.9885	0.0272	0.0220	0.9788	0.0298	0.0246	0.9882	0.0269	0.0217
	LSTM	0.9882	0.0275	0.0219	0.9677	0.0368	0.0294	0.9862	0.0292	0.0229
	GRU-LSTM	0.9877	0.0280	0.0225	0.9658	0.0379	0.0298	0.9854	0.0299	0.0238

**Table 4:** Cross-validation results.

Type	Model	Type		
		R^2^	RMSE (MPa)	MAE (MPa)
UCS	CNN-GRU-Attention	0.9417 ± 0.0231	0.5308 ± 0.0813	0.4369 ± 0.0718
	CNN-LSTM-Attention	0.9387 ± 0.0232	0.5455 ± 0.0868	0.4474 ± 0.0763
	CNN-BiLSTM-Attention	0.9363 ± 0.0245	0.5552 ± 0.1020	0.4525 ± 0.0894
	LSTM	0.8882 ± 0.0287	0.7440 ± 0.0729	0.6126 ± 0.0595
	GRU-LSTM	0.8842 ± 0.0293	0.7580 ± 0.0790	0.6216 ± 0.0688
STS	CNN-GRU-Attention	0.9329 ± 0.0242	0.0614 ± 0.0103	0.0496 ± 0.0086
	CNN-LSTM-Attention	0.9306 ± 0.0313	0.0622 ± 0.0108	0.0504 ± 0.0094
	CNN-BiLSTM-Attention	0.9323 ± 0.0233	0.0620 ± 0.0106	0.0503 ± 0.0095
	LSTM	0.8767 ± 0.0353	0.0836 ± 0.0094	0.0684 ± 0.0091
	GRU-LSTM	0.8739 ± 0.0356	0.0847 ± 0.0098	0.0694 ± 0.0091

## Data Availability

The original contributions presented in this study are included in the article. Further inquiries can be directed to the corresponding author.
